# Exploring diversity and inclusion in surgery: Insights from the 2025 Dutch Surgical Society Symposium

**DOI:** 10.1016/j.sopen.2025.08.002

**Published:** 2025-08-28

**Authors:** Begüm Pekbay, Hajar Rotbi, Michael El Boghdady, Joanna W.A.M. Bosmans, Joris J. Blok, Future Surgeon Initiative, Geneviève Koolhaas-Martis, Mahsa Montazeri, Abdelali Bentohami, Bernard Elsman, Kak Khee Yeung, Heleen S. Snijders, Diederik van Bakel, Floortje Huizing

**Affiliations:** 1 Consultant General Practitioner, Speaker and Moderator on Equity, Diversity, Inclusion in Healthcare, Utrecht, the Netherlands; 2 Department of Surgery, Maasstad Ziekenhuis, Rotterdam, the Netherlands; 3 BentoClinics, Amsterdam, the Netherlands; 4 Department of Surgery, Deventer Ziekenhuis, Deventer, the Netherlands; 5 Department of Surgery, Amsterdam University Medical Center, Amsterdam, the Netherlands; 6 Department of Surgery, Proctos Kliniek, Amsterdam, the Netherlands; 7 Department of Surgery, Franciscus Gasthuis & Vlietland, Rotterdam, the Netherlands; 8 Department of Surgery, Haaglanden Medical Center, The Hague, the Netherlands; a Department of Surgery, Erasmus Medical Center, Rotterdam, the Netherlands; b Faculty of Medicine, Radboud University Medical Center, Nijmegen, the Netherlands; c Department of Surgery, The Royal Marsden Hospital, London, United Kingdom; d University of Edinburgh, Edinburgh, United Kingdom; e Department of Surgery, IJsselland Ziekenhuis, Capelle aan den IJssel, the Netherlands; f Department of Surgery, Groene Hart Ziekenhuis, Gouda, the Netherlands; g Department of Surgery, Leiden University Medical Center, Leiden, the Netherlands

**Keywords:** Diversity, Health communication, Inclusion, Open surgical culture, Surgery

## Abstract

Diversity and inclusion are increasingly recognized as critical components of effective surgical practice and team dynamics. Despite growing awareness, structural barriers continue to limit the entry and advancement of individuals from underrepresented groups in surgery. At the annual meeting of the Dutch Surgical Society in 2025, a dedicated symposium on diversity and inclusion was organized to explore the relevance, urgency, and potential impact of these topics in surgery. Drawing from lived experience, research evidence, and a multidisciplinary panel discussion, this event underscored the necessity of shifting from performative diversity toward meaningful inclusion. Key insights from the symposium and actionable strategies to foster sustainable change within surgical education and practice are discussed in this paper. Future directions would include concrete policy development by the Dutch Surgical Society to achieve sustainable change, incorporating these topics in national education programs and development of structured mentorship networks and facilitating a continuous open dialogue.

## Introduction

In recent years, diversity has received increasing attention in surgery as means to improve both the working and learning environment [1]. However, while surgical teams may appear diverse on the surface, lack of inclusion, behavioral misalignment, or communication barriers can still undermine team performance and patient safety [2,3]. Research regarding diversity and inclusion within the field of surgery in the Netherlands is lacking.

## Setting the stage: defining diversity and inclusion in surgery

The Dutch Surgical Society (DSS) (Dutch; NVvH) is the national association for surgeons and surgical trainees in the Netherlands. It aims to advance the surgical profession through standard-setting, education, training, and research initiatives. The DSS serves as a central platform for surgical collaboration and development across the country. The annual congress, the ‘*Chirurgendagen*’, attracts over 2000 surgeons, surgical trainees, and researchers and provides a key opportunity for scientific exchange, professional networking, and critical discussions on the future of surgery in the Netherlands.

At the Chirurgendagen 2025, a dedicated symposium entitled “*Diversity and Inclusion: Are We There Yet?!*” was organized to discuss relevant topics in the field of diversity and inclusion. The symposium was initiated by the Diversity & Inclusion Working Group of the DSS in collaboration with the Future Surgeon Initiative [4]. The aim was to examine where we currently stand on diversity and inclusion in surgery, identify gaps, and spark a call to action. A panel of experts on the topic was formed who took the lead in the panel discussion with the attending participants. Topics and statements were formulated beforehand to discuss during the symposium.

The symposium began with a video introduction [5] and an oral presentation that provided context for diversity and inclusion in surgical practice. Diversity, in this context, is not merely demographic representation — it also encompasses differences in thought, background, work style, and values. Inclusion, on the other hand, is the deliberate practice of valuing and welcoming those differences, and ensuring psychological safety. As articulated in the symposium, “*Diversity is a fact. Inclusion is a choice. Culture is something we create together every day*.”

Next, gaps in recruitment, promotion, and leadership representation across gender and ethnic lines were highlighted, based on previously formulated statements. Empirical evidence suggests that individuals with a migration background remain significantly less likely to enter surgical training programs in the Netherlands (OR 0.55 [0.43–0.71], *p* < 0.001) [6]. Data from Statistics Netherlands (CBS) further highlighted this disparity: among the 1667 registered general surgeons, 77.3 % had no migration background [6]. This ‘leaky pipeline’ persists despite increased awareness, and systemic challenges continue to marginalize minorities diverse perspectives [7]. This results in unequal career progression and compromises the ability of the surgical field to serve a diverse student and patient population [[8], [9], [10], [11]].

The panelists were invited to discuss three statements based on their experiences navigating surgical training and practice (Table 1). The panel was composed to reflect diversity in age, gender, and professional roles, including two female and two male surgeons. This composition enabled a broad range of perspectives and enriched the discussion with lived experiences from the Dutch surgical landscape. The audience was then stimulated to join the discussion, nourishing a safe atmosphere in which individuals felt secure to share their personal and emotional experiences. In the presence of the current president of the DSS, the symposium concluded with a collective call to action in fostering more awareness on individual and systemic bias, and stimulating research regarding diversity and inclusion in surgery to develop concrete methods and tools for improvement.

**Table 1:** Panel discussion statements and summary of responses on diversity and inclusion in surgery.

Statements	General responses
A homogeneous surgical culture provides better discipline and patient safety than a diverse one.	Safety and discipline stem not from homogeneity, but from shared trust, clear communication, and inclusive leadership. Diversity contributes to these when inclusion is present. Transparency, open dialogue, and awareness of implicit norms help create equitable workplaces where every team member can thrive.
The surgical profession will become more attractive by closing the “leaky pipeline.” This starts with the surgical trainers.	Mentorship, equitable evaluation, and training in bias recognition are critical roles for surgical trainers yet it should be a shared responsibility within the whole department. Culture change should start at the level of surgical training programs, preferably earlier.

## Reflections and recommendations

The rationale for diversity and inclusion in surgery extends beyond equity. A diverse and inclusive surgical workforce is essential to ensure safe, empathetic, and patient-centered care. Ultimately, promoting diversity and inclusion is not only a moral imperative but a professional one, vital to the sustainability and excellence of our field. As we reflect on the symposium and the associated discussions, we highlight lessons learned with recommendations, supported by literature, to assist in concretizing strategies to foster diversity and inclusion in surgical teams.

## Key lessons learned

Firstly, diversity and inclusion are not static goals but dynamic, evolving responsibilities. Societal norms, patient populations, and surgical culture continuously shift—our definitions and strategies regarding diversity and inclusion must be adaptive accordingly [12]. Secondly, combatting unconscious bias begins with its acknowledgment and discussing existing dogmas [13]. By encouraging open dialogue and intersectionality, implicit assumptions can be reduced that otherwise hinder diversity and inclusion in surgical practice and decision-making [14,15]. Thirdly, diversity and inclusion should be reflected in leadership positions and surgeons can play a key role in organizational policy-making committees [16,17]. Diversity and inclusion policies should move beyond reaching certain percentages to sincere actions. Fourthly, surgical educators play a critical role in shaping future generations. Institutions must support them with the tools, training, and accountability to foster equitable mentorship, fair evaluations, and inclusive learning environments [18,19]. Lastly, establishing regular assessments of demographic trends and workplace culture ensures accountability [20]. Transparent data can identify gaps and track progress, informing targeted interventions to improve diversity and inclusion in surgery [21].

## Future directions

The Dutch surgical field is still in the early stages of meaningfully embedding diversity and inclusion in its long-term policy. While structural improvements have begun, such as a dedicated working group and attention at the national conference, actual cultural change is still not yet implemented. Moving forward, concrete policy development is the key to achieve sustainable long-term change. The DSS and affiliated surgical training centers should institutionalize diversity and inclusion goals, integrate these into surgical education, and create opportunities for reflection, feedback, and accountability. Educational modules and practical workshops can highlight the impact of bias, communication barriers, and inclusive teamwork on patient safety and surgical training. These modules can be incorporated in the national education programs for surgical trainees. The DSS can also actively confront the ‘leaky pipeline phenomenon’ by fostering leadership representation and ensuring representation of individuals with diverse backgrounds on surgical committees and training boards. Furthermore, establishing structured mentorship networks can support underrepresented medical students and trainees. Pairing them with surgical role models and mentors may help in advancing equality in surgical training and career options. Ultimately, maintaining the current momentum and facilitating open dialogue are essential for creating a widely accepted norm of diversity and inclusion within the current surgical culture.

In conclusion, the 2025 DSS symposium “*Are We There Yet?!*” demonstrated that meaningful change in the surgical culture begins with critical self-reflection and deliberate action. The open culture and safe environment to share personal experiences added emotional and meaningful depth to the symposium. By embracing diversity and inclusion as a core professional value, the surgical field can unlock its full potential to deliver safe, empathetic, and equitable patient care. A first step to go from paper to practice is starting with an open dialogue on these important topics, for example by implementing diversity and inclusion in the national surgical education programs, development of structured mentorship networks and facilitating a continuous open dialogue.

## CRediT authorship contribution statement

**Begüm Pekbay:** Writing – original draft, Investigation, Conceptualization. **Hajar Rotbi:** Writing – original draft. **Michael El Boghdady:** Writing – original draft. **Joanna W.A.M. Bosmans:** Writing – review & editing. **Joris J. Blok:** Writing – review & editing, Writing – original draft, Supervision, Conceptualization. **Geneviève Koolhaas-Martis:** Conceptualization. **Mahsa Montazeri:** Conceptualization. **Abdelali Bentohami:** Conceptualization. **Bernard Elsman:** Conceptualization. **Kak Khee Yeung:** Conceptualization. **Heleen S. Snijders:** Conceptualization. **Diederik van Bakel:** Conceptualization. **Floortje Huizing:** Conceptualization.

## Ethical approval

Ethics approval is not applicable.

## Authorship

All the authors have seen and agreed to the submitted version of the paper. All authors made substantial contributions to conception and design of the manuscript; drafting the article and revising it critically for important intellectual content; and all authors have approved the final version of the manuscript.

## Funding

No funding sources are to report.

## Declaration of competing interest

None of the authors have a conflict of interest to declare.
